# The dynamic nature and territory of transcriptional machinery in the bacterial chromosome

**DOI:** 10.3389/fmicb.2015.00497

**Published:** 2015-05-21

**Authors:** Ding J. Jin, Cedric Cagliero, Carmen M. Martin, Jerome Izard, Yan N. Zhou

**Affiliations:** Transcription Control Section, Gene Regulation and Chromosome Biology Laboratory, National Cancer Institute, National Institutes of HealthFrederick, MD, USA

**Keywords:** RNA polymerase, bacterial nucleolus, replisome, chromosome territories, growth rate regulation, stress responses, superresolution imaging, *E. coli*

## Abstract

Our knowledge of the regulation of genes involved in bacterial growth and stress responses is extensive; however, we have only recently begun to understand how environmental cues influence the dynamic, three-dimensional distribution of RNA polymerase (RNAP) in *Escherichia coli* on the level of single cell, using wide-field fluorescence microscopy and state-of-the-art imaging techniques. Live-cell imaging using either an agarose-embedding procedure or a microfluidic system further underscores the dynamic nature of the distribution of RNAP in response to changes in the environment and highlights the challenges in the study. A general agreement between live-cell and fixed-cell images has validated the formaldehyde-fixing procedure, which is a technical breakthrough in the study of the cell biology of RNAP. In this review we use a systems biology perspective to summarize the advances in the cell biology of RNAP in *E. coli*, including the discoveries of the bacterial nucleolus, the spatial compartmentalization of the transcription machinery at the periphery of the nucleoid, and the segregation of the chromosome territories for the two major cellular functions of transcription and replication in fast-growing cells. Our understanding of the coupling of transcription and bacterial chromosome (or nucleoid) structure is also summarized. Using *E. coli* as a simple model system, co-imaging of RNAP with DNA and other factors during growth and stress responses will continue to be a useful tool for studying bacterial growth and adaptation in changing environment.

## Features of E. coli genome that are important for cell growth and chromosome replication

### Genome, gene, and growth rate regulation

*Escherichia coli* cells, such as the prototype K-12 strain MG1655, are small rod-shaped, gram-negative bacteria. The *E. coli* genome contains ~4.6 million base pairs (bp). If fully stretched, a single *E. coli* genomic DNA is ~1600 μm long, ~1000-fold longer than the length of the cell; therefore the genome must be fully compacted to fit into a cell. The genome encodes 4453 genes, which are organized into about 2390 operons (Blattner et al., 1997; Riley et al., 2006). Not all genes are equal in terms of growth (or growth rate) regulation: *E. coli* genes can be broadly categorized in two functional classes: growth-promoting genes, represented by ribosomal RNA (rRNA) operons (for simplicity hereafter called *rrn*) and other genes.

The bacterial growth rate is determined by the growth medium (Kjeldgaard et al., 1958; Schaechter et al., 1958). Growth-promoting genes are few in number and mainly involved the synthesis of the translationary machinery, primarily *rrn* that encodes different species of rRNA and tRNA. Synthesis of rRNA is a rate-limiting step for the production of ribosomes (Gausing, 1980), as ribosomes are assembled onto nascent rRNAs. The number of ribosomes in the cell is proportional to the growth rate, which is needed to meet the demand for protein synthesis (Bremer and Dennis, 1996; Keener and Nomura, 1996). Because of the important role of rRNA synthesis in growth-rate regulation, the regulation of *rrn* has been extensively studied (Condon et al., 1995; Gourse et al., 1996; Wagner, 2002; Paul et al., 2004; Potrykus et al., 2011; Jin et al., 2012; Ross et al., 2013). The promoters of *rrn* are the most actively transcribed, accounting for >80% of total RNA synthesis in cells growing in nutrient-rich media (Bremer and Dennis, 1996), but become marginal under poor growth conditions or by the treatment of serine hydroxamate (SHX), a serine analog that triggers amino acid starvation (Tosa and Pizer, 1971) and induces the stringent response (Cashel et al., 1996). In addition, transcription of *rrn* is also regulated by an antitermination system containing NusA and NusB as well as other factors. While NusA binds to RNAP *in vitro* (Greenblatt and Li, 1981), NusB does not. NusB is thought to bind *in vivo* to the BoxA RNA sequences of nascent rRNA molecules and is also involved in rRNA processing (Torres et al., 2004; Bubunenko et al., 2013). Another difference between the two functional classes of genes is their respective genomic DNA content (see below): while the seven *rrn* operons (each ~5.5 kb in length) represent only ~1% of genomic DNA, other genes represent 99% of the genome.

### Bacterial growth and chromosome replication

**Figure 1:**
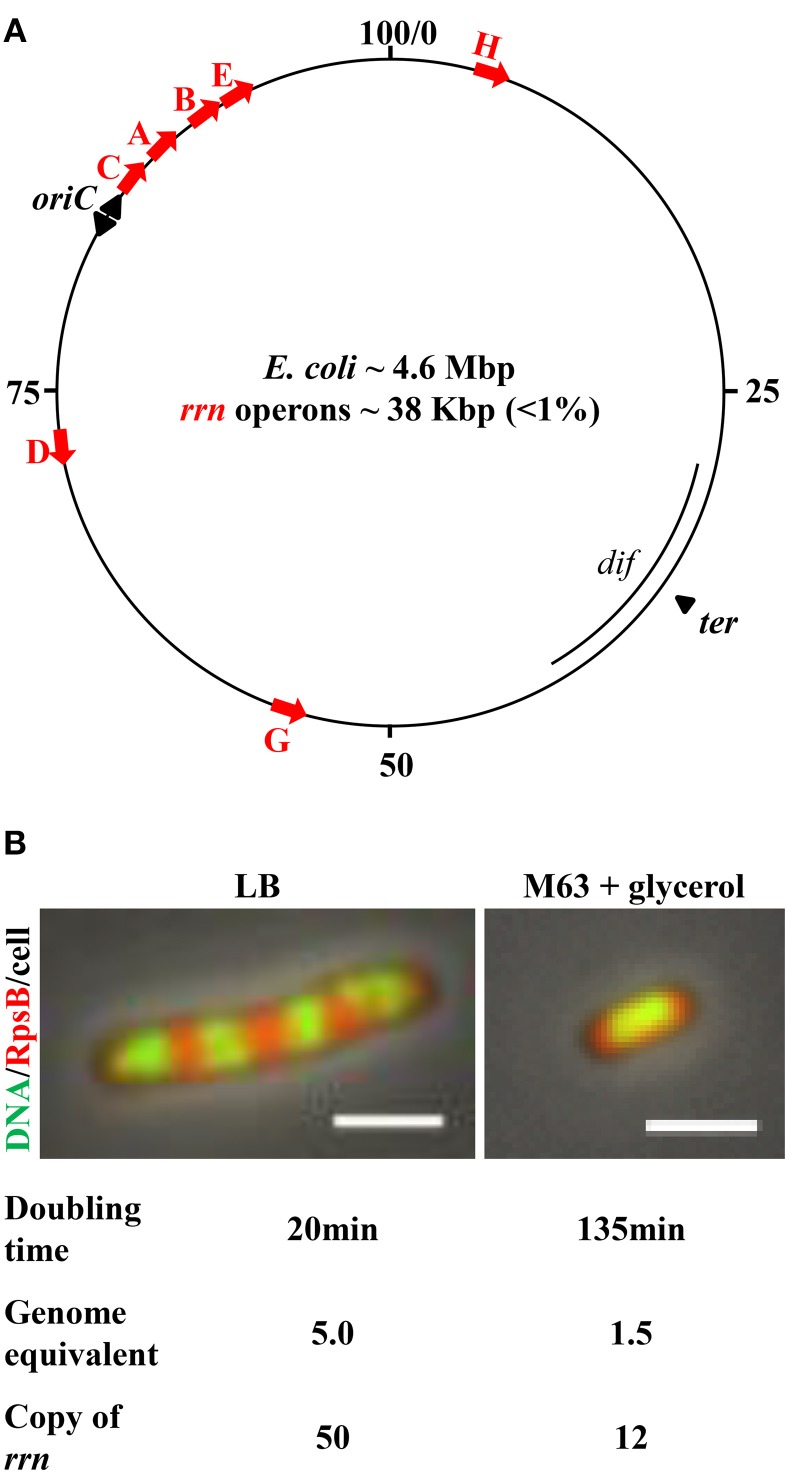
***E. coli*** **chromosome map and features important for growth rate regulation and genome replication**. **(A)** The genetic map of *E. coli* K-12. Positions of the *rrn* operons, along with *oriC*, and *ter* regions, are indicated. The red arrows represent each of the seven *rrn* operons, all of which are localized in the *oriC* proximal half of the chromosome; four are near the *oriC*, from which replication initiates and elongates bidirectionally, as indicated by black arrowheads. Transcription of *rrn* occurs in the same direction as replication. **(B)** Cell size and the number of genome equivalents and the *rrn* gene copies in a cell are sensitive to growth rate, as determined by growth medium. Images are overlays of nucleoid (green)/ribosome (red)/cell. Exponential-phase cells were prepared for imaging as described (Cabrera and Jin, 2003b). Ribosomes are tracked by 30S ribosomal subunit protein S2 fused with mCherry at the carboxy terminus (*rpsB*-*mCherry*). The nucleoid (DNA) is stained with Hoescht 33342. The cell growing in nutrient-rich LB (37°C) is large and has four apparent nascent nucleoids surrounded by ribosomes. The cell growing in minimal M63 + glycerol (30°C) is small and has only one apparent nucleoid surrounded with ribosomes. The scale bar represents 2 μm.

The *E. coli* genetic map is shown in Figure 1A. The chromosome is a circular DNA molecule with a specific origin of chromosome replication (*oriC*). After initiation, DNA replication proceeds bidirectionally as a pair of replication forks toward the terminus region (*ter*). As an integral component of replisomes (Yao and O'Donnell, 2010), single-stranded DNA binding protein (SSB) (Reyes-Lamothe et al., 2008; Marceau et al., 2011) coats the single-stranded DNA at the replication forks and interacts with the DNA polymerase III holoenzyme (O'Donnell, 2006). SeqA protein polymerizes with the nascent hemimethylated DNA at or near the DNA replication forks (Slater et al., 1995; Yamazoe et al., 2005; Waldminghaus et al., 2012).

Several features related to the *E. coli* lifestyle and the location of growth-promoting genes in the genome are important with respect to bacterial growth and chromosome replication.

First, the cell size and the copy number of the bacterial chromosome in a cell are sensitive to growth rate (Jin et al., 2013) (Figure 1B). The combined time required to complete a round of replication and subsequently chromosome segregation and cell division varies from ~70 to 150 min, depending on growth conditions (Stokke et al., 2012). Consequently, there are 1.5 genome equivalents (displaying one nucleoid surrounded by ribosomes) in a small slow-growing cell (doubling time 135 min) in M63 nutrient-poor media, containing glycerol as the carbon source at 30°C. When a cell's doubling time is shorter than the combined time required for replication and segregation, multiple initiations from *oriC* occur in preceding generations, manifesting “multi-fork replication” to ensure that at least one completed genome is passed onto each of the two newly formed daughter cells (Cooper and Helmstetter, 1968). For example, in a cell growing in nutrient-rich Lennox Broth (LB) at 37°C with a doubling time of ~20 min, the cell is large and there are up to five genome equivalents (displaying four nascent nucleoids surrounded by plentiful of ribosomes), and 8–16 copies of the *oriC* (Nielsen et al., 2007).

The second feature is the location of the seven *rrn* operons. All seven are located in the *oriC* half of the genome, and four of these are close to *oriC*. Because of the unique location of *rrn* relative to *oriC*, the gene dosage of *rrn* magnifies as the growth rate increases. For example, it can be calculated, based on the growth rate and the location of the *rrn* in the genetic map (Condon et al., 1993; Bremer and Dennis, 1996), that when cells are growing in LB at 30°C, with a doubling time of 45 min, the copy number for *rrn* is 18 per cell with more than two genome equivalents; however, with a doubling time of 20 min (LB, 37°C), the copy number of *rrn* approaches 50 per cell. This feature is particularly significant, as it dictates the differential allocation of RNAP between *rrn* and other genes genome-wide in response to changes in the environment, as described below. In addition, the transcription of *rrn* and most of the components of the translational machinery is in the same direction as that of chromosome replication (Rocha and Danchin, 2003; Lin et al., 2010). This co-directionality has likely evolved to minimize replication–transcription conflicts in bacteria (Merrikh et al., 2012).

## Differential allocation of RNA polymerase between *rrn* and other genes genome-wide in response to a changing environment: evidence from genetic and genomic studies

### Competition for limited RNAP between *rrn* and other genes genome-wide in fast-growing cells

It becomes evident that RNAP is limiting in the cell for the simultaneous active expression of *rrn* and the broad transcription of other genes genome-wide. This notion was first suggested by the study of a class of RNAP mutants that altered growth rate regulation: these mutants grew slowly in LB because of reduced transcription of *rrn*, as if they were partially starved for amino acids, even in nutrient-rich medium. In contrast, the expression of some other genes that are activated only during starvation in wild type was elevated in those mutants grown in LB (Zhou and Jin, 1997, 1998). Combined with the biochemical studies of these RNAP mutants, it was proposed that RNAP is limiting in the cell for simultaneous active transcription of *rrn* and other genes involved in the stress response and thus RNAP redistributes from *rrn* to other genes in the genome during the stringent response induced by amino acid starvation (Zhou and Jin, 1998). Analysis of these RNAP mutants motivated the initiation of research on the cell biology of RNAP as described below.

RNAP is primarily allocated to the active transcription of *rrn* in cells growing in nutrient-rich media. Micrographs of chromatin spreads of fast-growing cells revealed that RNAP molecules are packed during *rrn* synthesis, at an estimated 65 RNAP molecules per *rrn* (French and Miller, 1989). The estimated number of RNAP molecules in a cell varies by quantitative Western blot analysis (Ishihama, 2000; Grigorova et al., 2006; Piper et al., 2009), but direct counting of single molecules using photoactivated localization microscopy (PALM) yields an independent measurement of ~5000 RNAP molecules in a fast-growing cell (Bakshi et al., 2012; Endesfelder et al., 2013). Consequently, in a fast-growing cell with a doubling time of 20 min (LB, 37°C), active transcription of the estimated 50 copies of *rrn* will consume 3250 RNAP molecules, indicating that ~65% of the total RNAP (assuming ~5000 molecules per cell) is allocated for rRNA synthesis. To use an analogy, in fast-growing cells, most of the bacterial RNAP functions as eukaryotic Pol I/III activities to promote growth. In eukaryotic cells Pol I is responsible for the synthesis of rRNA at the nucleolus (O'Sullivan et al., 2013) and Pol III makes small RNAs including tRNAs. Consequently, only a small fraction of *E. coli* RNAP engages in transcription of other genes in the remaining ~99% of the genome, consistent with the finding that most regions of the chromatin spreads appeared to be “naked,” or devoid of RNAP (French and Miller, 1989).

### Redistribution of RNAP from *rrn* to other genes genome-wide during stress responses

When fast-growing cells are exposed to nutrient limitation/starvation or to stresses, the immediate response of cells is to “turn off” *rrn* expression, leading to the decrease and/or arrest of growth, and concomitantly to “turn on” other genes involved in the response. The “feast or famine” or “thrive or survive” lifestyle not only reflects the amazing ability of *E. coli* to rapidly respond and/or adapt to changing environments but also represents the two ends of the spectrum in bacterial growth rate regulation. Global transcriptional profiling of cells grown on different carbon sources has indicated that there is an apparent inverse correlation between the growth potential of the environment and the number of genes expressed systematically genome-wide (Liu et al., 2005). The poorer the quality of carbon source used, the larger the number of genes that are expressed genome-wide. Similarly, when fast-growing cells are subject to amino acid starvation with the addition of SHX, leading to the stringent response (Cashel et al., 1996), transcription of *rrn* becomes minimal and the cells' growth arrests, whereas the number of other genes expressed across the genome increases systematically (Durfee et al., 2008; Traxler et al., 2008). To use another analogy, in cells undergoing stress responses, most of the bacterial RNAP functions as eukaryotic Pol II activities for bulk mRNA synthesis at the expense of the *rrn* expression.

This apparent inverse relationship between the expression of *rrn* and the number of other genes expressed genome-wide in response to changes in the environment indicates that growth regulation and stress responses can be explained by genome-wide competition between *rrn* and other genes for limited RNAP in the cell (Jin et al., 2012). The differential allocation of RNAP between the two functional classes of genes in response to changes in the environment also argues against the notion that there are a significant number of “inactive” RNAP molecules in the cell (Bremer and Dennis, 1996).

Extensive studies with different approaches have pointed to the special role of rRNA synthesis in both cell growth and the competition for the limited RNAP in the transcription of other genes genome-wide in response to environmental cues. These studies have provided an intellectual foundation for understanding the cell biology of RNAP in cells under different growth conditions.

## Imaging the dynamic distribution of RNAP in cells responding rapidly to environmental cues: challenge and a breakthrough solution

### The distribution of RNAP in fast-growing cells undergoes continuous change during sampling preparation and live-cell imaging

Shortly after green fluorescent protein (GFP) technology was introduced into bacteria (Gordon et al., 1997; Lewis et al., 2000; Margolin, 2000), an *E. coli* chromosomal *rpoC-gfp* fusion as the RNAP-GFP reporter was constructed to study the cell biology of RNAP in fast-growing cells and in cells undergoing an amino acid starvation–induced stress response (Cabrera and Jin, 2003a,b). Since then, many derivatives of the RNAP-GFP reporter have been constructed in *E. coli* to study the distribution of RNAP under different conditions using wide-field microscopy and other cutting-edge imaging techniques (Cabrera et al., 2009; Bakshi et al., 2012; Cagliero and Jin, 2013; Endesfelder et al., 2013; Jin et al., 2013).

**Figure 2:**
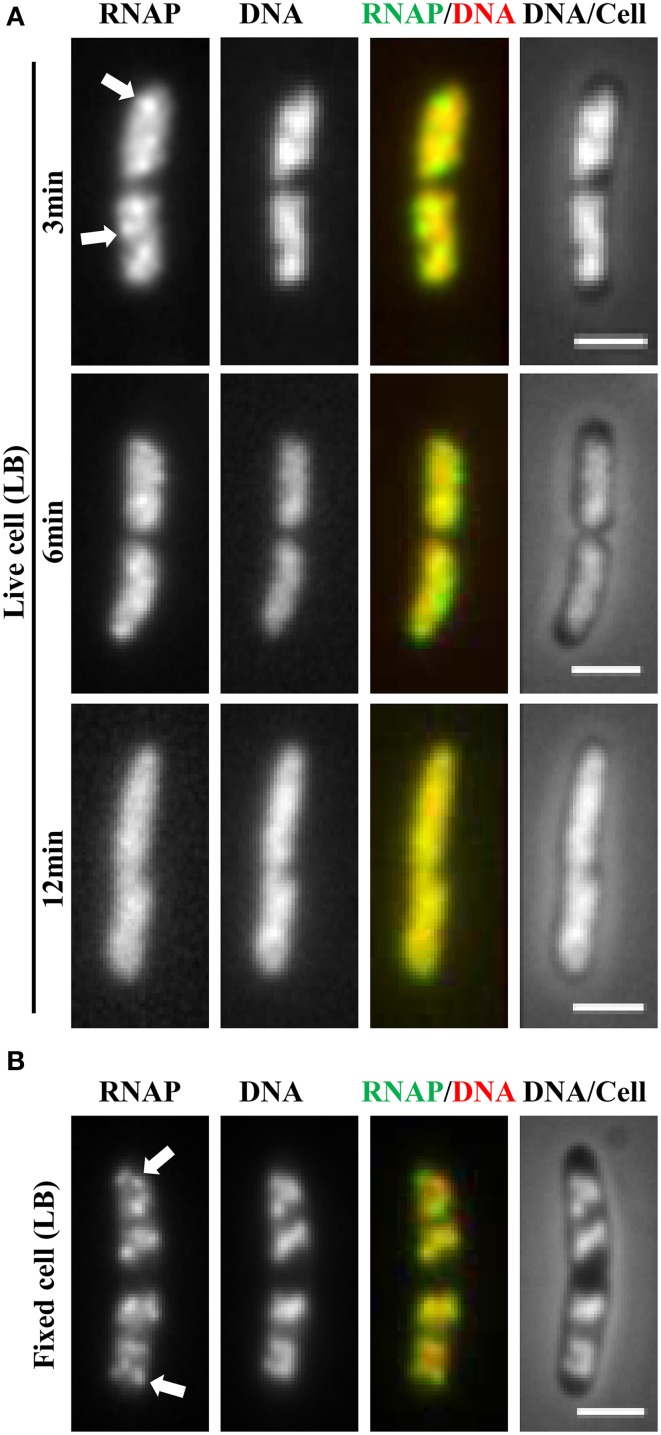
**Challenge of imaging RNAP in fast-growing living cells and a breakthrough technique using a formaldehyde-fixed cell procedure**. **(A)** Rapidly disappearing RNAP foci during sampling and imaging of fast-growing living cells (LB, 37°C). Exponential-phase cells (*rpoC-venus*) in a rapidly shaking flask in a water bath were sampled and agarose embedded on coverslips for imaging at different time intervals as indicated. The sampling and preparation were performed as described (Cabrera and Jin, 2003b), except that there was no formaldehyde treatment. The nucleoid (DNA) was stained with Hoescht 33342. **(B)** Same as in **(A)**, except that the cells were fixed with formaldehyde immediately after sampling. RNAP foci were stable in the formaldehyde-fixed cells for a few hr before imaging. Foci are indicated by arrows. The scale bar represents 2 μm.

**Figure 3:**
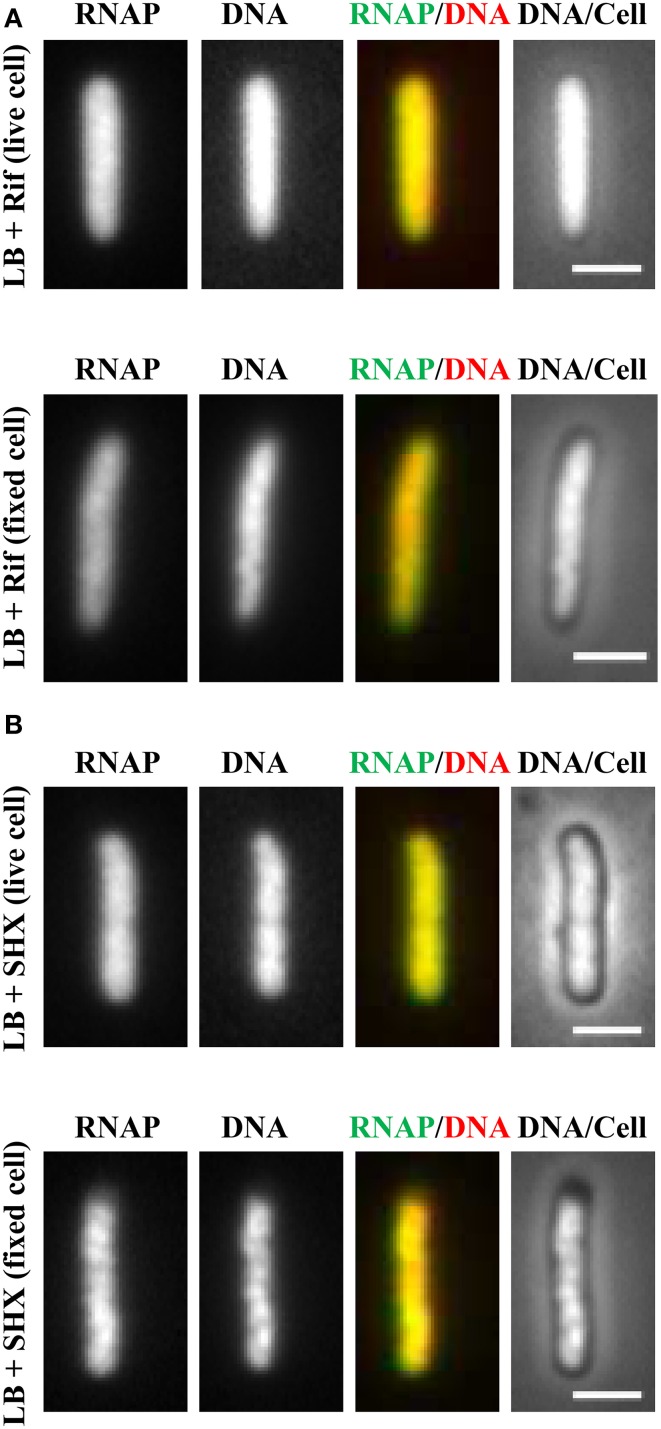
**Inhibition of** ***rrn*** **expression leads to homogeneous distribution of RNAP across the nucleoid in both living and fixed cells**. Fast-growing cells (LB, 37°C) were treated with either rifampicin (100 μg/ml) for 15 min **(A)** or with SHX (500 μg/ml) for 30 min **(B)**. Cells were sampled and agarose embedded on coverslips. Live-cell imaging (live cell); Fixed-cell imaging (fixed cell), cells were fixed by formaldehyde. The scale bar represents 2 μm.

It soon became evident that the distribution of RNAP is extremely sensitive to physiological perturbations, including those caused by the sampling and imaging processes; such a property has not been reported for other GFP reporters, such as the *parS*-ParB-GFP system (Nielsen et al., 2007) and the SSB-GFP fusion used in tracking the replisome (Reyes-Lamothe et al., 2008). Initially, no difference was observed in the RNAP distribution in living cells under different growth conditions. Figure 2A shows a set of images of living cells growing in a rapidly shaking flask (LB, 37°C), a condition in which most RNAP molecules are engaged in active rRNA synthesis. These cells were imaged after sampling from the flask and then agarose-embedded on coverslips at room temperature (25°C). The pattern of RNAP distribution changes depending on the length of time between sampling preparation and imaging. For example, RNAP foci could be observed in some cells after a short time (3 min); however, as the length of time increases (6 min), only few cells have RNAP foci, and RNAP is homogeneously distributed in the nucleoid in the cell population after 12 min. Note that nucleoid structure also changes in parallel and becomes expanded as time increases during the imaging process, indicating also the dynamic nature of the nucleoid. Experimentally, the time elapsed between sampling and image acquisition is usually longer than 10 min. The same distribution pattern was also observed in fast-growing cells treated either with rifampicin (Figure 3A, living cell), or with SHX (Figure 3B, living cell), which inhibit *rrn* transcription. RNAP is distributed homogeneously in the nucleoid regardless of growth conditions and status of *rrn* synthesis in living cells under the conditions used.

### Using formaldehyde-fixed cells revolutionizes the imaging of RNAP

Realizing that fast-growing cells in a rapidly shaking flask quickly adapt to slow-growth or growth arrest on coverslips during the sampling procedure, which usually takes between 10 and 15 min, and that sequential imaging would capture only the adapted states of RNAP, thereby missing the “true” state of RNAP in fast-growing cells, it became essential to develop a new sampling procedure. To this end, a formaldehyde-fixed cell procedure was developed, in which cells under different physiological conditions are immediately fixed or cross-linked with formaldehyde to “freeze” RNAP and subcellular structures before embedding them in agarose on coverslips for imaging. Formaldehyde is a cell-permeable, small four-atom molecule that penetrates cells rapidly and cross-links stably bound proteins to DNA (Schmiedeberg et al., 2009). This breakthrough technique has enabled the capture of dynamic states of RNAP in *E. coli* as snapshots in changing environments; the results have been shown to be consistent with the genetics and physiology of *E. coli* cells (Cabrera and Jin, 2003b). For example, RNAP forms prominent foci (Figure 2B, fixed cell) at the clustering of *rrn*, resembling a bacterial nucleolus in fast-growing cells (LB, 37°C) (more below). In contrast, there are no RNAP foci in cells treated with either rifampicin (Figure 3A, fixed cell) or SHX (Figure 3B, fixed cell); instead, RNAP distributes homogeneously across the nucleoid. These images have provided the first biological evidence, at a single-cell level, of the redistribution of RNAP from the clustering of *rrn* in fast-growing cells to a broad genome-wide transcription (or RNAP binding) in cells undergoing stresses. Intriguingly, changes in the distribution of RNAP accompany changes in nucleoid structure (see Figures 2, 3), revealing the role of RNAP and transcription in the organization of the bacterial chromosome (Jin and Cabrera, 2006; Jin et al., 2013) (more below). On a cautionary note, the dynamic nature of the nucleoid, which responds to changes in the environment and sampling and imaging processes, should be taken into consideration when studying cell cycles using live-cell imaging.

### Live-cell imaging using continuous-flow microfluidics has further validated the use of formaldehyde in the study of the cell biology of RNAP

**Figure 4:**
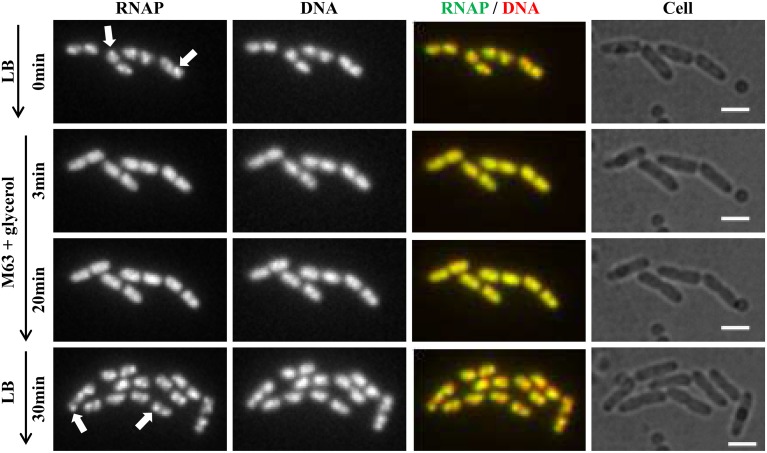
**RNAP foci are evident in fast-growing cells and dynamic in response to nutrient downshift and upshift using live-cell imaging with continuous-flow microfluidics**. Cells (*rpoC-venus, hupA-mCherry*) were growing in a microfluidic device controlled by the CellASIC ONIX microfluidic perfusion system (EMD Millipore) with continuous flow of LB. After exponential-phase cells in LB were imaged (LB, 0 min), LB was replaced with M63 + glycerol, and time-lapse images were taken, two of which, at 3 min and 20 min, are shown. At 20 min, the minimal medium was replaced with LB, and 30 min later, the image shown was taken. The experiments with microfluidics were performed at 30°C because LB has high background autofluorescence at 37°C. It took about 1 min to complete a medium change in the system. Hu-mCherry was used as a proxy for DNA staining because the microfluidic device used has high autofluorescence in the range of 460–488 nm, which interferes with the detection of DNA-bind dye DAPI or Hoescht 33342. The scale bar represents 2 μm. RNAP foci are indicated by arrows. Note the changes in the cells' position and size during the imaging process.

**Figure 5:**
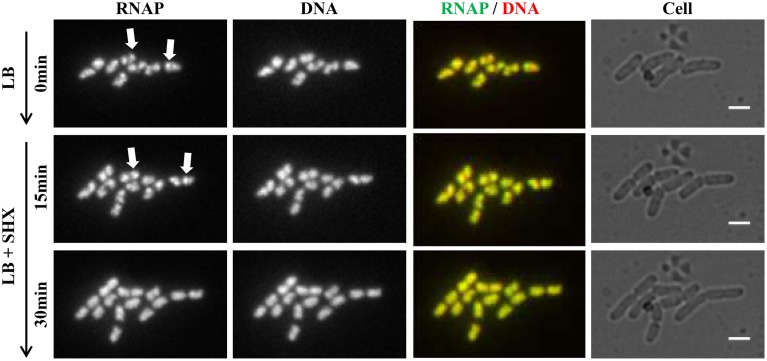
**Live-cell imaging using microfluidics confirms that RNAP foci disappear in cells treated with SHX, causing amino acid starvation**. Cells (*rpoC-venus, hupA-mCherry*) were grown in LB using the CellASIC ONIX microfluidic system, as described in Figure 4. After exponential-phase cells in LB were imaged (LB, 0 min), LB was replaced with LB + SHX (500 μg/ml), and cells were imaged at different intervals after the SHX treatment. Images taken at 15 and 30 min after the addition of SHX are shown. The scale bar represents 2 μm. RNAP foci are indicated by arrows. Note the changes in the cells' position and size during the imaging process.

**Figure 6:**
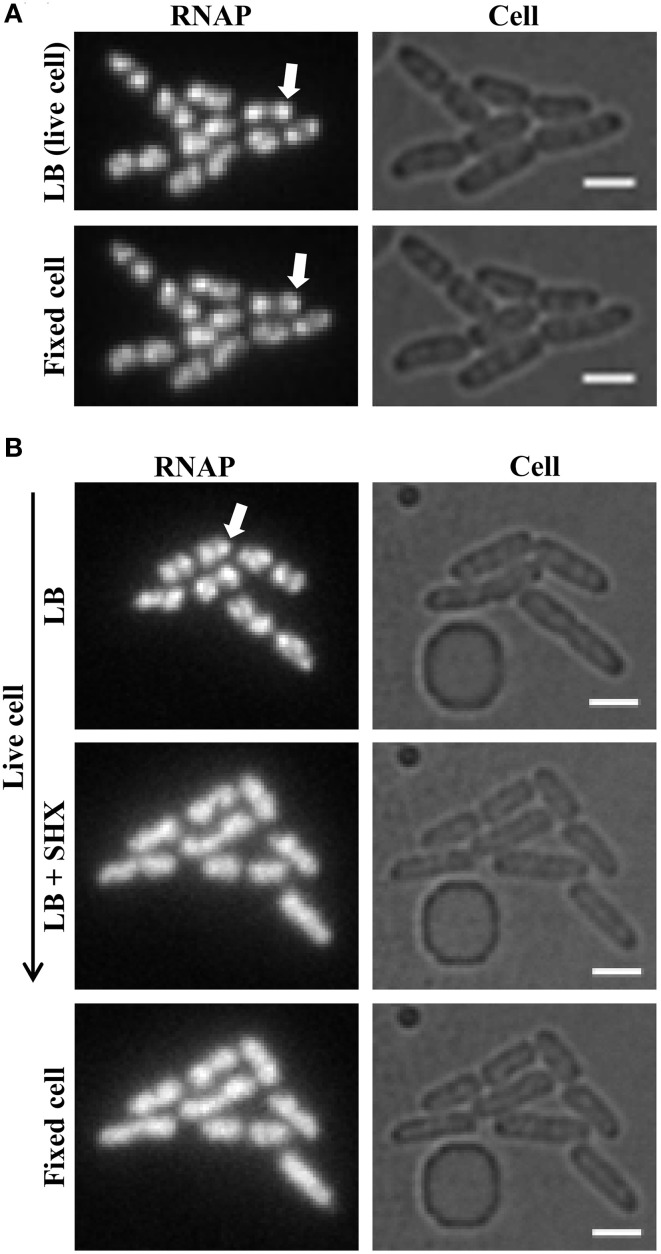
**Formaldehyde freezes the dynamic states of RNAP in living cells**. **(A)** RNAP foci in fast-growing cells are preserved by formaldehyde treatment. Live-cell imaging with continuous-flow microfluidics was performed as described in Figure 4. After exponential-phase cells in LB were imaged (LB, live cell), LB was replaced with LB + formaldehyde (final concentration 3.7% v/v) for 10 min to fix the cells, followed by flushing with M63 + glycerol for 10 min before imaging (fixed cell). **(B)** Homogenous distribution of RNAP across the nucleoid is maintained in amino acid-starved cells by formaldehyde treatment. The early steps of live-cell imaging, including LB and LB + SHX for 30 min to induce amino acid starvation, were performed as described in Figure 5. The subsequent formaldehyde treatment steps were performed as described in **(A)** for fixed-cell imaging. For simplicity, only images of RNAP and cells are shown. RNAP foci are indicated by arrows. The scale bar represents 2 μm.

Recently, continuous-flow microfluidics has been introduced for live cell imaging of *E. coli* (Wang et al., 2010). This technique has the advantage of enabling continuous live-cell imaging of RNAP in fast-growing and/or in changing environments without sampling interruptions. Time-lapse images from a set of such experiments are shown in Figure 4. RNAP foci are evident in fast-growing living cells in LB (RNAP and RNAP/nucleoid overlay), and the foci disappear shortly (~3 min) after the culture is downshifted from LB (at time 0) to nutrient-poor, minimal medium, M9 + glycerol; the process is reversible, as RNAP foci reappear after the culture is upshifted back to LB (RNAP and RNAP/Nucleoid overlay). Treatment of fast-growing cells in LB with SHX for 30 min (Figure 5) causes the RNAP foci to disappear (RNAP and RNAP/Nucleoid overlay) almost completely. In addition, RNAP foci are preserved after formaldehyde treatment of fast-growing cells in microfluidics (Figure 6A), whereas RNAP maintains a homogenous distribution pattern after the formaldehyde treatment of cells that were starved for amino acid by the SHX treatment (Figure 6B). Together, these findings demonstrated that formaldehyde does not cause “artificial” perturbations in the organization of RNAP and DNA in cells, as has been shown in ChIP-chip assays (Davis et al., 2011), and that the distribution of RNAP from fixed-cell images (Cabrera and Jin, 2003b, 2006; Cabrera et al., 2009; Cagliero and Jin, 2013; Endesfelder et al., 2013; Jin et al., 2013), reflects the true dynamic states of RNAP in living cells, thus validating the use of formaldehyde in the study of the cell biology of RNAP.

Imaging of RNAP in formaldehyde-fixed cells has significant advantages over live-cell imaging. Living cells, particularly fast-growing cells, undergo high metabolic activities, such as DNA replication and cell division, which constantly generate internal motions and may also change cells' positions in microfluidics during the imaging process (Figures 4, 5). Also, because LB has high background autofluorescence, the quality of RNAP live-cell images, particularly in fast-growing cells in microfluidics, is rather poor. In addition, for some studies, the physiology of cells in a continuous-flow microfluidic device may be different from that of cells in shaking flasks in a water bath. In contrast, formaldehyde-fixed cells are immobile, and this characteristic is critical and mandatory for acquiring sharp superresolution co-images of RNAP and DNA, as demonstrated below.

It is noteworthy that the formaldehyde-fixed cell procedure has also been widely used in ChIP-chip assays to study the distribution of *E. coli* RNAP genome-wide in one dimension (Grainger et al., 2005; Herring et al., 2005; Davis et al., 2011) and in various chromosome conformation capture assays to probe chromosome organization in living cells (Dekker et al., 2002; Dostie et al., 2006; Umbarger et al., 2011; Cagliero et al., 2013). It is expected that the formaldehyde-fixed cell procedure will also be useful for the capture of dynamic states of other cellular machineries in *E. coli*.

## Chromosome territories in *E. coli*: a landscape of transcription machinery and replisome

### Significance of the study of the cell biology of RNAP

Significant progress has been made in imaging the distribution of RNAP in *E. coli* in response to changing environments and these studies have shed new light on the differential allocation of RNAP in during bacterial growth and stress responses. Findings from the images of RNAP at the single-cell level complement other genomic analyses of transcription and RNAP binding, including gene arrays and ChIP-chip assays, which are used in population studies and in which the data involved in rRNA synthesis or RNAP binding at the *rrn* regions are purposely deleted to “simplify” the analyses. Consequently, these analyses have missed a significant part of the whole story in the lifestyle of *E. coli*, as transcription of *rrn* plays a key role in growth rate regulation. Specifically, as described below, recent co-imaging of RNAP with DNA and components of the replisome in a single cell has revealed some important features related to the spatial organization of the transcription machinery as well as the landscape of the transcription machinery and replisome (Cagliero et al., 2014). Features of the chromosome territories in bacteria are analogous to those of eukaryotes (Cremer et al., 2006; Meaburn and Misteli, 2007), indicating that *E. coli* is a useful simple model system to study chromosome biology in response to changes in the environment.

### Bacterial nucleolus

**Figure 7:**
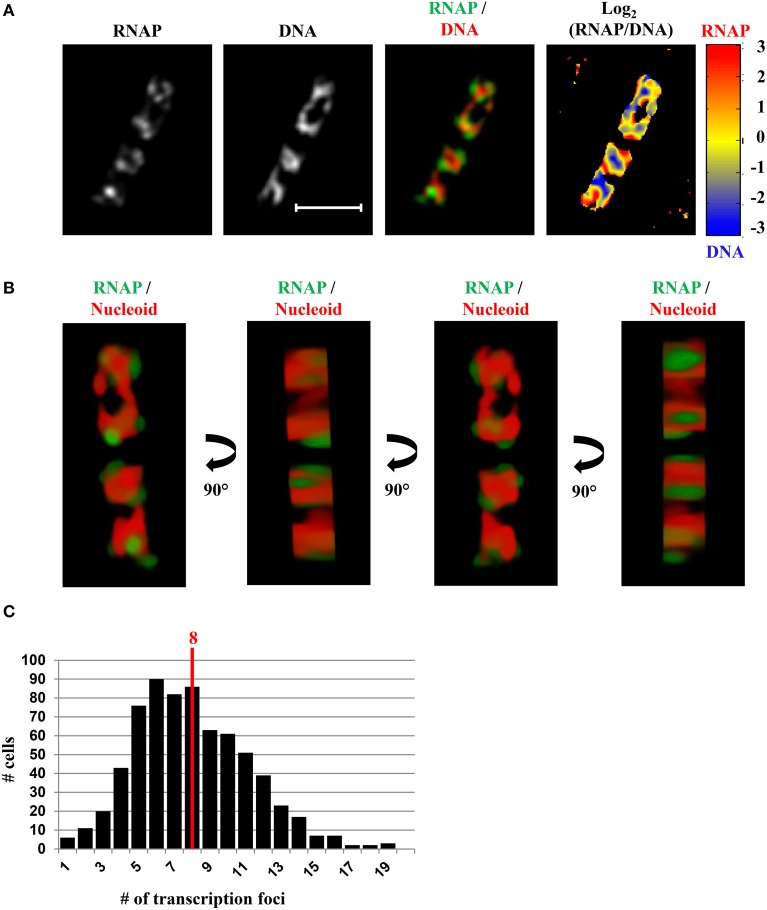
**SIM co-imaging of RNAP and DNA reveals spatial compartmentalization of transcription foci in fast-growing cells. (A)** Images of RNAP, DNA (nucleoid), and an overlay of RNAP (green) and DNA (red) from a representative fast-growing *E. coli* cell (LB, 37°C). The scale bar represents 2 μm. The log_2_(RNAP/DNA) plot (heat map) is a quantitative representation of the relationship between RNAP and DNA, which is represented by a color scale bar with values ranging from −3 to 3. Note that regions enriched in RNAP up to eightfold over DNA are at the periphery of the nucleoid (red foci), and regions enriched in DNA up to eightfold over RNAP are in the center of the nucleoid (blue regions). **(B)** 3D representation of the overlay image of RNAP (green) and nucleoid (DNA) (red) in the cell, as shown in **(A)**. Compared with the first and the third panels (x–y axis), the second and fourth panels reflect the limited Z-stacking due to the short axis of the cell. **(C)** Histogram showing the distribution of apparent RNAP-Venus foci in fast-growing cells. The red line indicates the median number of transcription foci in the population of cells. Modified and adapted from Cagliero et al. (2014).

In fast-growing cells, most RNAP molecules are concentrated, forming foci at the clustering of *rrn*, which resembles the eukaryotic nucleolus. The presence of the bacterial nucleolus is inferred from the finding using wide-field microscopy, that the number of RNAP foci is significantly lower than the number of copies of *rrn* in a fast-growing cell (Cabrera and Jin, 2003b). Subsequent super-resolution images have further confirmed this finding (Endesfelder et al., 2013; Cagliero et al., 2014). For example, super-resolution structured illumination microscopy (SIM) of RNAP and DNA (Cagliero et al., 2014) (Figure 7) reveals that the median number of RNAP foci is eight per cell in fast-growing cells (LB at 37°C, doubling time 20 min) (Figure 7C). Compared with the estimated average of 50 *rrn* copies, then, each RNAP focus occurs, on average, at approximately six copies of *rrn*. The resolution (~140 nm lateral and ~300 nm axial for a typical Venus fluorophore) of SIM would be as effective as PALM in detecting small transcription foci in fast-growing cells. Indeed, analysis of PALM images reveals that even small clusters (foci) of 70 RNAPs form a sphere of ~160 nm in diameter (Endesfelder et al., 2013), which is larger than the resolution detectable by SIM. Whether *rrn* from different locations in the chromosome are clustered with RNAP foci remain to be determined experimentally.

There are up to four nascent nucleoids in a fast-growing cell; thus, on average, there are two RNAP foci per nascent nucleoid in the cell. The size of RNAP foci in fast-growing cells varies, probably reflecting the dynamic nature of the foci, as they are likely undergoing disassembly and reassembly when replication forks pass through the *rrn* region. The size variation of RNAP foci is consistent with the super-resolution images, using PALM, of both large clusters of RNAP (up to 800 molecules), which are likely engaging in rRNA synthesis from multiple *rrn* operons, and small clusters of RNAP (~70 molecules), which are attributed to the transcription of a single *rrn* operon (Endesfelder et al., 2013). As each nascent nucleoid has RNAP foci in place, each of the two newly formed daughter cells will “inherit” the functional transcription machinery without the need for *de novo* synthesis and/or assembly. Because transcription foci at the bacterial nucleolus are critical for growth rate regulation in *E. coli* (Jin et al., 2012), this feature can explain, in part, why newly formed daughter cells maintain the same fast growth rate as the parental cells.

### Compartmentalization of RNAP foci at the periphery of the nucleoid

A significant advantage of co-imaging RNAP with DNA is that it enables the determination of the spatial relationship between the transcription machinery and DNA. In a fast-growing cell, RNAP foci appear to be located at the periphery or surroundings of the nucleoid (RNAP/DNA overlay), either in two-dimensional SIM pictures (Figure 7A) or reconstituted three-dimensional SIM images (Figure 7B). The quantitative heat map, as a normalized Log_2_(RNAP/DNA) density plot, further illustrates an inverse relationship between the intensities of RNAP and DNA (Figure 7A), indicating that RNAP foci at the clustering of *rrn* are low in DNA density or at loops at the surface of the nucleoid. Such a spatial organization has logistical advantages, including coupling of rRNA synthesis with rRNA processing and ribosome assembly, as well as reducing the traffic jams with other cellular functions.

RNAP foci are likely to be networking hubs for transcription of growth-promoting genes, because genome conformation capture assays have shown that those genes, which are down-regulated during amino acid starvation, are in a highly interactive environment and are present in large clusters (Cagliero et al., 2013). The spatial compartmentalization of RNAP foci suggests that transcription foci could be used as RNAP pools to effectively interact with DNA loops containing non-*rrn* growth-promoting genes located a distance away by hopping three-dimensionally, rather than by traveling linearly or laterally (Wang et al., 2013). Such an organization would reduce the transcription traffic jam and allow for maximum use of limited RNAP in the cell (Jin et al., 2012). It would be interesting to identify other non-*rrn* genes and RNAs as well as proteins in these potential transcription hubs. Other subcellular hyperstructures in bacteria have been described (Norris et al., 2007).

### Components of *rrn* antitermination and rRNA processing systems are colocalized with RNAP foci

**Figure 8:**
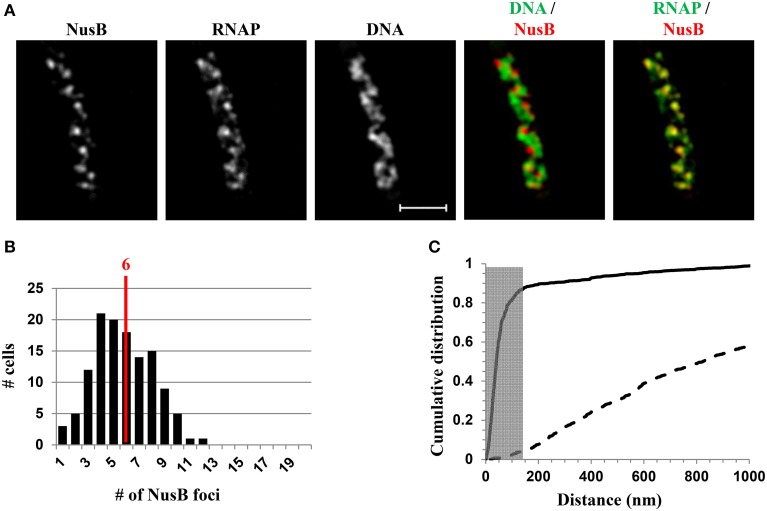
**Nascent rRNA-binding protein NusB forms foci and colocalizes with transcription foci in fast-growing cells**. **(A)** Images of NusB, RNAP, DNA (nucleoid), overlay of NusB (red) and DNA (green), and overlay of RNAP (green) and NusB (red) from a representative fast-growing *E. coli* cell, as described in the legend to Figure 7. NusB foci are at the periphery of the nucleoid (separate red and green colors on the RNAP/NusB overlay) and the NusB signals perfectly colocalize with RNAP signals (overall yellow color on the RNAP/NusB overlay). **(B)** The distribution of apparent NusB-mCherry foci in fast-growing cells. The red line in the histogram indicates the median number of NusB foci in the population of cells. Note that the median number of NusB foci is close to that of RNAP foci in fast-growing cells. **(C)** Cumulative distribution of the distances between NusB foci and their closest RNAP foci in the population of cells. (—-) NusB-mCherry RNAP-Venus, and (- - -) NusB-mCherry RNAP-Venus random. The gray rectangle represents the colocalization area (≤140 nm), as the theoretical SIM microscope resolution for the mCherry is 140 nm. 87.1% of the NusB foci are within 140 nm of the closest transcription foci. Adapted from Cagliero et al. (2014).

NusA and NusB are involved in *rrn* antitermination and rRNA processing systems. SIM co-imaging of RNAP-Venus with DNA and NusA-mCherry or NusB-mCherry in fast-growing cells has demonstrated that, (i) like RNAP, NusA, or NusB forms foci at the periphery of the nucleoid, and the median number of NusA or NusB foci per cell is similar to that of the RNAP foci, and (ii) the NusA or NusB foci are co-localized with RNAP foci (Cagliero et al., 2014). For example, Figure 8 shows the SIM images of NusB and its spatial relationship with DNA (DNA/NusB overlay) and with RNAP (RNAP/NusB) in a typical fast-growing cell (LB at 37°C) (Figure 8A). There were six NusB foci per cell on average in a population of fast-growing cells (Figure 8B), a value that is close to that of RNAP foci. The cumulative distribution of NusB foci and RNAP foci from a population of fast-growing cells has confirmed that most of NusB foci (>87%) are colocalized with RNAP foci at the clustering of *rrn* or bacterial nucleolus (Figure 8C), demonstrating that rRNA synthesis and processing are intimately coupled in space. It remains to be determined what other components are associated with transcription foci in fast-growing cells.

### Spatial segregation of transcription machinery and replisome

Unlike a eukaryotic cell that has defined phases in the cell cycle (S, G2, M, and G1), all processes–such as transcription, replication, and chromosome segregation, are intimately entangled in a rapidly growing *E. coli* cell. Therefore, maximum expression of growth-promoting genes and multiple genome replications are occurring concurrently in a fast-growing cell. A long-standing interest in the field relates to understanding how the two major cellular functions, transcription and replication, maintain harmony to avoid conflicts between the two processes (Merrikh et al., 2012), particularly in fast-growing cells.

**Figure 9:**
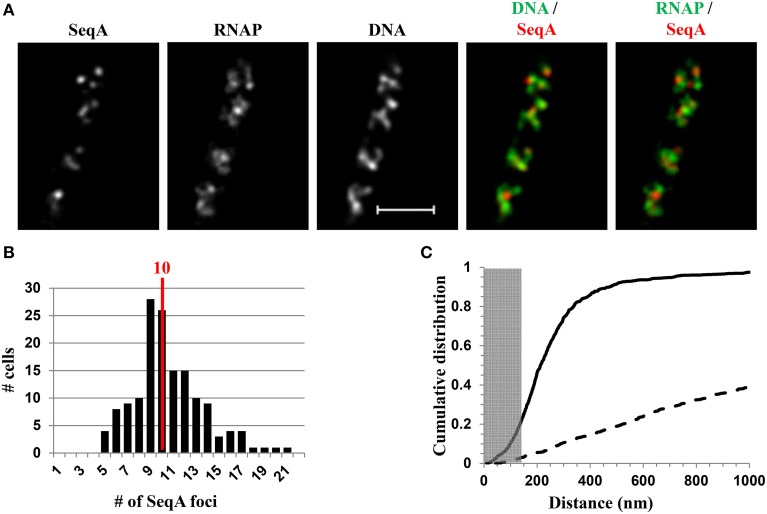
**Spatial segregation of transcription foci and replication forks tracked by SeqA in fast-growing cells**. **(A)** Images of SeqA, RNAP, DNA (nucleoid), overlays of SeqA (red) and DNA (green), and RNAP (green) and SeqA (red) from a representative fast-growing *E. coli* cell, as described in the legend to Figure 7. SeqA foci and transcription foci are largely located at different positions (red and green colors on the overlay of SeqA and RNAP). Note also that most of the SeqA foci appear to be separated from high intensities of DNA signals in the nucleoids (red and green colors on the overlay of SeqA and DNA). **(B)** The distribution of apparent SeqA-mCherry foci in a population of fast-growing cells. The red line in the histogram indicates the median number of SeqA foci in these cells. **(C)** Cumulative distribution of the distances between SeqA foci and their closest RNAP foci in the population of cells. (—-) SeqA-mCherry RNAP-Venus, and (- - -) SeqA-mCherry RNAP-Venus random. The gray rectangle represents the colocalization area (≤140 nm). Only 21.5% of the SeqA foci are within 140 nm of the closest transcription foci. Adapted from Cagliero et al. (2014).

Recent SIM co-images of RNAP-Venus with DNA as well as with SeqA-mCherry or SSB-mCherry serving as proxies for replisomes have revealed chromosome landscapes for the two major functions of transcription and replication, which could explain why they remain in harmony in fast-growing cells. The major transcription machinery and replisome are mostly located in different chromosome territories or spatially segregated in the nucleoid (Cagliero et al., 2014). For example, Figure 9A shows the SIM images of SeqA and its spatial relationship with DNA (DNA/SeqA overlay) and with RNAP (RNAP/SeqA) in a typical fast-growing cell (LB at 37°C). Similar to RNAP foci and foci of NusA/NusB, SeqA foci also are located at the periphery of the nucleoid (DNA/SeqA overlay), indicating that the replisome is also compartmentalized in regions low in density of DNA or at DNA loops. Image analyses of populations of fast-growing cells showed that on average, each cell contains 10 SeqA foci (Figure 9B). In contrast to NusA/NusB, the cumulative distribution of SeqA foci and RNAP foci in the population of fast-growing cells has shown that most (~80%) of the SeqA foci are not colocalized with the RNAP foci, i.e., the two cellular functions are mostly segregated in space (Figure 9C). The low co-localization frequency of SeqA foci and RNAP foci suggests transient overlapping of transcription and replication of *rrn* regions. It is conceivable that during replication of *rrn* operons, RNAP foci are somehow disassembled, allowing replication forks to pass through the region, followed by reassembly of transcription machinery at the *rrn* clusters. Development of fast, super-resolution, time-lapse, live-cell imaging techniques will be necessary to address the dynamic interaction and segregation of the two active cellular functions in fast-growing cells.

## Coupling the distribution of RNAP to the organization of the bacterial nucleoid

### Active rRNA synthesis at the clustering of *rrn* causes nucleoid compaction

**Figure 10:**
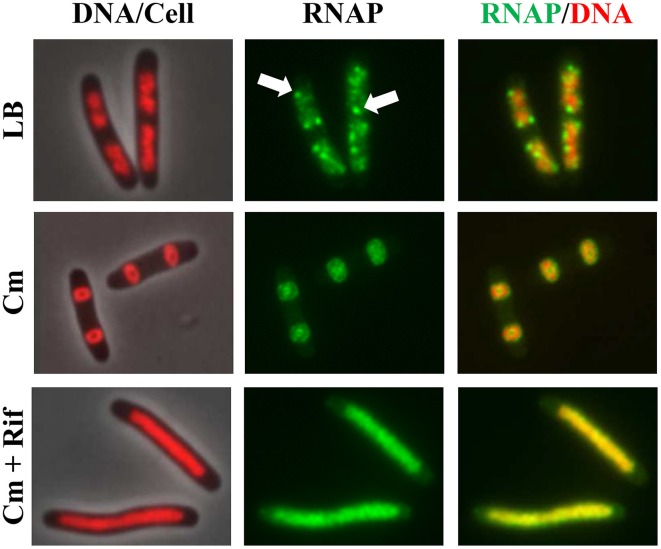
**Active** ***rrn*** **expression is required to condense the nucleoid in the presence of chloramphenicol**. Images of overlay of DNA (nucleoid) and cell, RNAP, and overlay of RNAP and DNA are shown. DNA in the DNA/cell overlay is shown in red (left column), RNAP is shown in green (middle column), and the RNAP (green) and DNA (red) overlay is shown (right column). The panels on the top row show rapidly growing cells (*rpoC-gfp*) in LB, at 30°C, and RNAP foci are indicated by arrows; panels in the center row show cells treated with chloramphenicol (100 μg/ml) for 100 min; panels in the bottom row show cells sequentially treated with chloramphenicol (100 μg/ml, 10 min) and rifampicin (100 μg/ml, 90 min). Modified and adapted from Cabrera et al. (2009).

Co-imaging of RNAP and DNA in cells undergoing physiological perturbations has also revealed that global changes in the distribution of RNAP accompany alterations in nucleoid structure, indicating an important role of RNAP and transcription in the organization of the bacterial chromosome (Jin and Cabrera, 2006; Jin et al., 2013). The changes are stress-dependent and in most cases studied, as shown in Figures 2, 3, redistribution of RNAP from a few prominent foci at the clustering of *rrn* to the nucleoid homogeneously leads to nucleoid expansion. In an effort to determine whether active rRNA synthesis is required to condense the nucleoid, the effects of the antibiotics chloramphenicol and rifampin as well as two mutations that decrease rRNA synthesis, on the nucleoid structure have been reexamined (Cabrera et al., 2009). The nucleoids are condensed in fast-growing cells (LB) treated with the translation inhibitor chloramphenicol (Woldringh et al., 1995; van Helvoort et al., 1996); however, if cells are treated sequentially with chloramphenicol first and then rifampicin, the nucleoids become expanded and RNAP foci disappear (Figure 10). Moreover, the nucleoids remain expanded in the above-described RNAP mutant cells defective in the transcription of *rrn* in LB when treated with chloramphenicol. Similarly, even with chloramphenicol treatment, the nucleoids remain expanded in the Δ6*rrn* cells that have only one copy of *rrn* remaining in the chromosome. Together, these results support the role of active rRNA synthesis from the clustering of *rrn* in nucleoid compaction in fast-growing cells. Because transcription and supercoiling are coupled (Liu and Wang, 1987), it is possible that transcription-induced supercoiling at the clustering of *rrn* (Jin et al., 2012) contributes to the nucleoid compaction in fast-growing cells.

The mechanisms underlying the apparent coupling of the distribution of RNAP with the organization of the bacterial chromosome remain to be determined. Multiple factors are likely to be responsible for the organization of the bacterial nucleoid (Woldringh et al., 1995; Cagliero et al., 2013; Jin et al., 2013). Other small nucleoid-associated proteins (NAPs) or “histone-like” proteins, such as FIS, H-NS, HU, and IHF, play important architectural roles and are transcription factors (Luijsterburg et al., 2006; Browning et al., 2010; Dillon and Dorman, 2010; Liu et al., 2010; Pul and Wagner, 2012). The distribution of RNAP will likely to be affected by these NAPs and vice versa; together, they determine the organization of the nucleoid.

### Temporal changes in the distribution of RNAP and the nucleoid structure in response to osmotic stress

**Figure 11:**
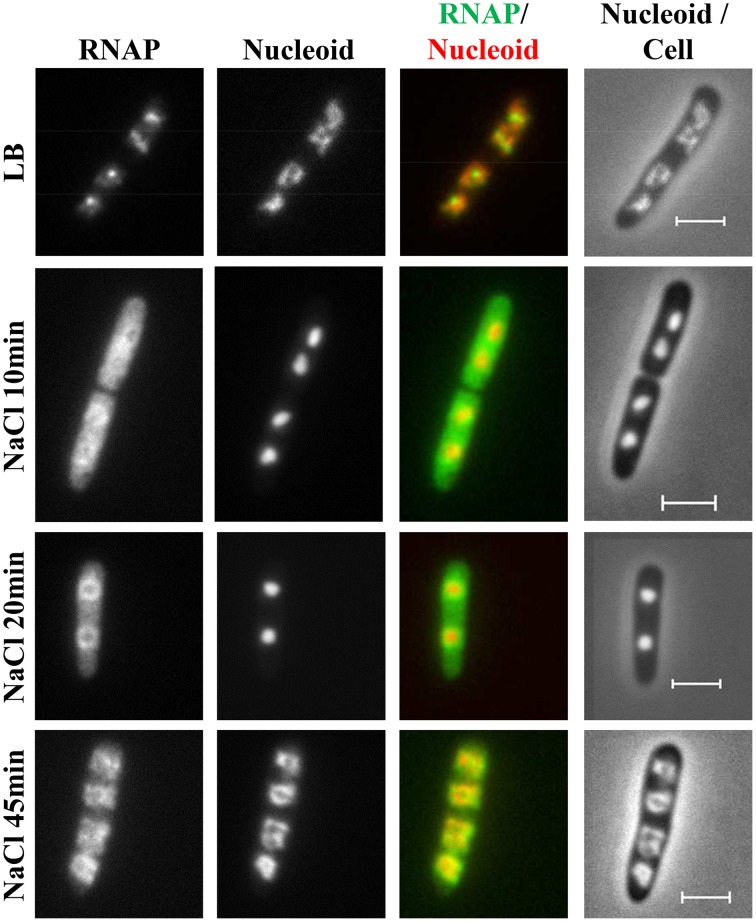
**Dynamic interaction of RNAP and DNA during the osmotic stress response**. Images of RNAP, nucleoid, overlays of RNAP (green) and nucleoid (red), and nucleoid and cell (*rpoC-venus*) before LB or after osmotic shock (NaCl 10 min to NaCl 45 min). The RNAP rapidly dissociates from the nucleoid, and the released RNAP occupies the entire cytoplasmic space shortly after osmotic shock (NaCl 10 min). The nucleoid becomes hyper-condensed at the same time. Subsequently, the freed RNAP gradually re-associates with the genome by first forming a ring at the periphery of the hyper-condensed nucleoid (NaCl 20 min to NaCl 30 min) before penetrating the interior (NaCl 45 min). The scale bar represents 2 μm. Adapted from Cagliero and Jin (2013).

During hyperosmotic stress response, the changes in RNAP distribution and nucleoid compaction are temporally manifested (Cagliero and Jin, 2013). Initially, significant numbers of RNAP molecules dissociate from the nucleoid into the cytoplasmic space because of the transient accumulation of the cytoplasmic K^+^, and, concomitantly, the nucleoid becomes hyper-condensed (Figure 11). Subsequently, when the cytoplasmic K^+^ levels decrease during the osmoadaptation phase, the free RNAP re-associates with DNA and initially forms a ring at the periphery of the nucleoid, and the nucleoid gradually expands to a size approaching that prior to the salt shock. The ring of RNAP surrounding the hyper-condensed nucleoid during the early osmoadaptation phase (NaCl 20 min) is thought to indicate the location of DNA loops for the expression of responsive genes. The causes of temporal changes of the nucleoid structure during the osmotic stress response are not clear.

## Summary

**Figure 12:**
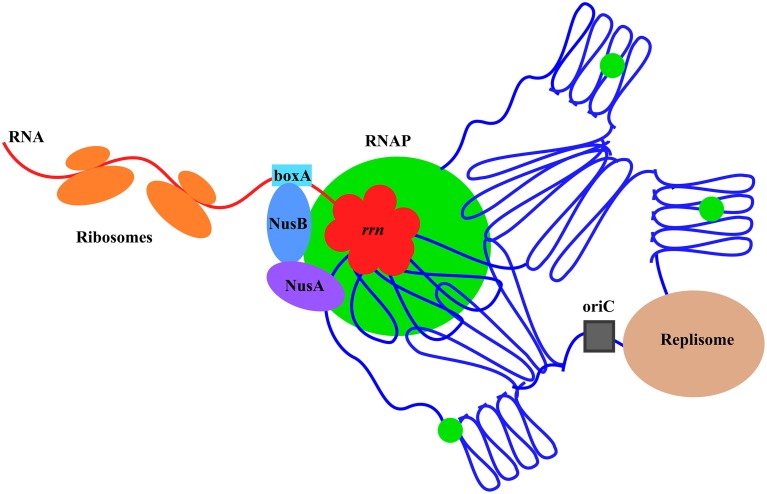
**Model illustrating the chromosome territories of the major transcription machinery and replisome in a fast-growing cell**. The *E. coli* chromosome is represented by the blue lines folded in loops, the *oriC* by a black square, and the RNAP molecules by green circles. For simplicity, only one of the prominent transcription foci and the replisome are shown. RNAP foci at the clustering of *rrn* (red), resembling a bacterial nucleolus, are spatially organized at the periphery of the nucleoid for compartmentalization. Foci of NusA and NusB co-localize with RNAP foci, indicating the coupling of the synthesis and processing of rRNA, and, possibly, ribosomal assembly. Compartmentalization of the replisome is also shown, but the two major cellular functions of transcription and replication are largely spatially segregated. See text for the details and advantages of the landscapes of transcription machinery and replisome in fast-growing cells.

Co-imaging of RNAP with DNA as well as with other proteins in cells under different growth conditions has been an important tool and a new approach in the long-standing journey to understanding growth rate regulation and stress responses in *E. coli*. Significant progress has been made, including new concepts and findings from these studies, particularly in fast-growing cells (Figure 12). It is expected that more will be learned using wide-field and state-of-the-art imaging systems in future studies. In addition, as a simple model system, *E. coli* will have many advantages in seeking fundamental knowledge of the chromosome biology including chromosome territories, a frontier in biology research.

### Conflict of interest statement

The authors declare that the research was conducted in the absence of any commercial or financial relationships that could be construed as a potential conflict of interest.
